# Fish Nutrition and Metabolism: From Intestinal Microbiota to Metabolic Homeostasis

**DOI:** 10.3390/metabo16060350

**Published:** 2026-05-23

**Authors:** Lei Wang, Peng Tan, Rantao Zuo

**Affiliations:** 1 School of Ecology and Environment, Anhui Normal University, Wuhu 241002, China; leiwang@ahnu.edu.cn; 2 Key Laboratory of Mariculture and Enhancement, Zhejiang Marine Fisheries Research Institute, Zhoushan 316021, China; 3 Key Laboratory of Mariculture and Stock Enhancement in North China’s Sea, Ministry of Agriculture and Rural Affairs, Dalian Ocean University, Dalian 116023, China

## 1. Introduction

Aquaculture continues to be a crucial sector for global food security. As intensive farming practices expand, optimising fish nutrition and metabolism is essential to ensure health, improve product quality, and promote industry sustainability. Historically, the evaluation of novel and sustainable feed formulations has relied heavily on traditional zootechnical parameters and body composition data. However, these classical indicators are often insufficient to elucidate the intricate ways in which distinct feedstuffs and nutrients dynamically modulate fish metabolism. Today, the intersection of holistic metabolic profiling, dietary strategies, and intestinal microecology has emerged as a primary focal point for understanding host physiological homeostasis [1,2]. Dietary interventions and alternative protein sources significantly influence the dynamic balance of the gut microbiota and metabolic pathways, directly dictating growth efficiency and disease resistance [3,4]. This Special Issue of *Metabolites*, “Fish Nutrition and Metabolism,” compiles six original research articles that explore the intricate relationships among formulated diets, rearing environments, intestinal health, and metabolic regulation across diverse aquatic species.

## 2. Special Issue Contribution

The first major theme of this Special Issue addresses the application of functional feed additives to modulate lipid and protein metabolism alongside intestinal microbiota. In aquaculture, ensuring efficient lipid utilisation is critical to prevent abnormal lipid deposition and associated hepatic metabolic disorders [5]. Zhang et al. (Contribution 1) investigated the inclusion of lysolecithin in stearin-based high-lipid diets for largemouth bass (*Micropterus salmoides*). Their integrated transcriptomic and microbiome analyses revealed that lysolecithin activated the PI3K-AKT signalling pathway, enhanced lipid mobilisation, and increased the relative abundance of beneficial gut bacteria, notably *Cetobacterium*. Similarly, Zhou et al. (Contribution 2) demonstrated that feeding yellow drum (*Nibea albiflora*) larvae with soybean lecithin-enriched *Artemia nauplii* significantly improved intestinal microvillus morphology and upregulated critical lipid catabolism genes, thereby promoting growth performance and desiccation stress resistance. Addressing the physiological challenges of low-fishmeal diets, Ren et al. (Contribution 3) showed that supplementing feeds with crayfish by-product protein hydrolysate effectively mitigated metabolic stress in Pacific white shrimp (*Litopenaeus vannamei*). This hydrolysate reversed the adverse transcriptional changes in appetite-related genes and reshaped the intestinal microbiota, highlighting the potential of bioactive peptides to maintain feeding motivation and gut homeostasis under low-fishmeal conditions.

The second theme focuses on how ecological rearing models, such as integrated rice−fish co-culture systems, impact host metabolism and flesh quality. Yan et al. (Contribution 4) evaluated the physiological benefits of rice-paddy environments for crucian carp (*Carassius auratus*). They observed that compared to traditional pond culture, rice−fish farming significantly enhanced hepatic antioxidant capacity and glucose metabolism enzymes, yielding muscle tissue with higher crude protein and lower crude lipid. Complementing these findings, Zhou et al. (Contribution 5) compared the muscle quality and intestinal microbiomes of hook snout carp (*Opsariichthys bidens*) raised in rice fields versus ponds. Their study identified distinct differences in volatile flavour profiles and gut microbiota assemblages, suggesting that external rearing environments shape muscle nutritional quality by altering the dynamic stability of the microbial community.

Finally, understanding fundamental metabolic changes during organismal ageing is vital for establishing comprehensive physiological models. Labeille et al. (Contribution 6) utilised an untargeted metabolomics approach to characterise bone ageing in the model fish Japanese medaka (*Oryzias latipes*). Their research identified sex-independent hyperglycosylation as a key indicator of chronological ageing, offering novel insights into the metabolic flux underlying skeletal senescence.

In conclusion, this Special Issue provides comprehensive insights into the nutritional and metabolic regulation of aquatic animals. The findings underscore the critical importance of integrating multi-omics approaches to decode the complex crosstalk between dietary inputs, the gut microbiome, and host metabolism. The findings underscore the critical importance of integrating multi-omics technologies, including 16S rDNA sequencing, non-targeted metabolomics, and transcriptomics, to map these dynamic metabolic networks comprehensively.

We extend our sincere gratitude to all the contributing authors, peer reviewers, and the editorial team of *Metabolites* for their dedication and professionalism. We hope these contributions will inspire further research into functional feeds and the intestine−liver metabolic axis, ultimately driving the sustainable evolution of the aquaculture industry.

## 3. Future Research Directions

The rapidly evolving field of fish nutrition and metabolism, particularly with the integration of high-throughput metabolomics, offers numerous compelling research opportunities. Based on the findings of this Special Issue and the current state of the art, several key areas warrant further investigation. For example, future studies should move beyond single-omics profiling to integrate metabolomics with metagenomics and transcriptomics. This holistic approach is essential to decode the complex “crosstalk” within the different organs/tissues in fish.

We extend an enthusiastic invitation to researchers to address these critical knowledge gaps and contribute to emerging areas of investigation. The forthcoming second edition of our Special Issue will focus on these themes and will welcome innovative studies that advance our understanding of fish nutrition and metabolism. We particularly welcome original research articles investigating the host−microbiota metabolic interaction and its impact on aquatic animal health. In addition, studies exploring precision nutrition strategies and targeted metabolic interventions, as well as methodological papers introducing innovative analytical approaches, such as multi-omics integration or non-invasive biomarker validation, are also warmly welcomed.
